# "Features of two proteins of Leptospira interrogans with potential role in host-pathogen interactions"

**DOI:** 10.1186/1471-2180-12-50

**Published:** 2012-03-30

**Authors:** Renan F Domingos, Monica L Vieira, Eliete C Romero, Amane Paldes Gonçales, Zenaide M de Morais, Silvio A Vasconcellos, Ana L T O Nascimento

**Affiliations:** 1 Centro de Biotecnologia, Instituto Butantan, Avenida Vital Brazil, 1500, 05503-900, São Paulo, SP, Brazil; 2 Pós-Graduação Interunidades em Biotecnologia, Instituto de Ciências Biomédicas, USP, Avenida Prof. Lineu Prestes, 1730, 05508-900, São Paulo, SP, Brazil; 3 Centro de Bacteriologia, Instituto Adolfo Lutz, Avenida Dr. Arnaldo, 355, CEP 01246-902, Sao Paulo, Brazil; 4 Laboratório de Zoonoses Bacterianas do VPS, Faculdade de Medicina Veterinária e Zootecnia, USP, Avenida Prof. Dr. Orlando Marques de Paiva, 87, 05508-270, São Paulo, SP, Brazil

## Abstract

**Background:**

Leptospirosis is considered a re-emerging infectious disease caused by pathogenic spirochaetes of the genus *Leptospira*. Pathogenic leptospires have the ability to survive and disseminate to multiple organs after penetrating the host. Leptospires were shown to express surface proteins that interact with the extracellular matrix (ECM) and to plasminogen (PLG). This study examined the interaction of two putative leptospiral proteins with laminin, collagen Type I, collagen Type IV, cellular fibronectin, plasma fibronectin, PLG, factor H and C4bp.

**Results:**

We show that two leptospiral proteins encoded by LIC11834 and LIC12253 genes interact with laminin in a dose - dependent and saturable mode, with dissociation equilibrium constants (*K*_D_) of 367.5 and 415.4 nM, respectively. These proteins were named Lsa33 and Lsa25 (Leptospiral surface adhesin) for LIC11834 and LIC12253, respectively. Metaperiodate - treated laminin reduced Lsa25 - laminin interaction, suggesting that sugar moieties of this ligand participate in this interaction. The Lsa33 is also PLG - binding receptor, with a *K*_D_ of 23.53 nM, capable of generating plasmin in the presence of an activator. Although in a weak manner, both proteins interact with C4bp, a regulator of complement classical route. *In silico* analysis together with proteinase K and immunoflorescence data suggest that these proteins might be surface exposed. Moreover, the recombinant proteins partially inhibited leptospiral adherence to immobilized laminin and PLG.

**Conclusions:**

We believe that these multifunctional proteins have the potential to participate in the interaction of leptospires to hosts by mediating adhesion and by helping the bacteria to escape the immune system and to overcome tissue barriers. To our knowledge, Lsa33 is the first leptospiral protein described to date with the capability of binding laminin, PLG and C4bp *in vitro*.

## Background

Leptospirosis is a zoonosis caused by pathogenic species of the genus *Leptospira*. Greater incidence of human infection occurs in tropical and subtropical countries [1,2]. The transmission of leptospirosis has been correlated with exposure of individuals in close proximity to wild or farm animals [1,3]. Recently, the disease became prevalent in cities with sanitation problems and large urban rodent reservoirs that contaminate the environment through their urine [4].

Pathogenic *Leptospira* spp. have ability to adhere and rapidly disseminate within the host during the early stage of infection. Surface - associated proteins are potential targets to mediate host - pathogen interactions, and therefore are likely to elicit several activities, including adhesion. The adhesion of leptospires to ECM components of the host was considered to be essential in the initial stage of the infection [5]. Indeed, we have reported that pathogenic leptospires are capable of binding several ECM molecules [6]. To date, several leptospiral ECM binding adhesins have been described [6-18]. After the adhesion, pathogens have to overcome tissue barriers in order to reach blood circulation and organs. We have reported that leptospires have the ability of binding PLG at their surface and that plasmin (PLA) can be generated in the presence of activator [19]. In addition, Verma and colleagues [20] and our group have described several leptospiral proteins as PLG - binding receptors [17,18,21]. More recently, we have reported that PLA generation on *Leptospira* decreased opsonization and that it might be an important aspect of the immune escape strategy and survival [22].

*L. interrogans* serovar Copenhageni genome annotation identified many unknown coding sequences predicted to be surface exposed proteins. Characterization of these proteins, with no previously assigned function, should increase our understanding of this intriguing pathogen’s biology. In this work, we present our studies with two leptospiral coding sequences, LIC11834 and LIC12253, named Lsa33 and Lsa25, respectively. The genes were cloned and the proteins expressed using *E. coli.* The recombinant proteins were purified and their ability to bind various ECM and serum components was evaluated. We report that these proteins are novel surface adhesins capable of binding to laminin. In addition, Lsa33 can also interact to PLG and both proteins bind the complement regulator of the classical pathway C4bp. We believe that these proteins are likely to be involved in *Leptospira -* host interactions.

## Results

### Bioinformatic analysis

The selected coding sequences, LIC11834 and LIC12253, are genome annotated as hypothetical proteins, and one of them, LIC11834, is a putative lipoprotein, having lipoprotein signal peptide (signal peptidase II) and a cleavage site between amino acids 17–18. According to SMART web server, LIC11834 has a signal peptide from 1 to 21 amino acids and a FecR domain from amino acid 60 to 162. PFAM predicts that this domain is involved in regulation of iron dicitrate transport and that FecR is probably a sensor that recognizes iron dicitrate in the periplasm. LIC12253 presents a signal peptide from amino acid 1 to 21 and a DUF1566 (Domain of Unknown Function) from amino acid 58 to 164 [23,24]. The LIC11834 coding sequence can be classified as alpha - beta protein, being the percentage of 36.57 for alpha-helix and 29.13 for beta strands secondary structure. In the case of coding sequence LIC12253, the protein can be classified as mixed, having a predicted secondary structure composition percent of 11.01, 19.38 and 69.60 for alpha - helix, beta strands and others, respectively. Cellular localization predicts as extra - cellular (non-cytoplasmic branch) for both proteins. The solvent accessibility composition (core/surface ratio) for the CDs LIC11834 and LIC12253 is expected to be 59.87 and 66.52% of amino acid residues exposed with more than 16% of their surface, respectively. All the predictions above were performed with PrecitedProtein web server [25,26]. The presence and identity of both coding sequences among *Leptospira* sequenced genomes are depicted in Table 1.

**Table 1:** Gene locus, given names, features, gene conservation, sequence of the primers employed for DNA amplification, and molecular mass of expressed recombinant proteins

**Gene locus**^**1**^	**Given name**^**2**^	**Description/** **Function**	**Conservation (identity)**^**3**^	**Sequence of primers for PCR amplification**	**Molecular mass**
LIC11834	Lsa33	Putative lipoprotein	Lai (99%) LBH (87%) LBP (31%)	F:5′CTCGAGGATCTACAAGGTGGGGTTTTTAC3′ XhoI R:5′CCATGGTTACTGAGGTTTTACTTGGTCC3′ NcoI	33.1 kDa
LIC12253	Lsa25	Conserved hypothetical protein	Lai (100%) LBH (77%) LBP (39%)	F:5′ CTCGAGGAGGAGAAACCGGACGATAC 3′ XhoI R:5′CCATGGTTAGGGAAGACTTCTAACACATC3′ NcoI	24.07 kDa

^1^http://aeg.lbi.ic.unicamp.br/world/lic/; LIC: *Leptospira interrogans* Copenhageni

^2^Lsa: Leptospiral surface adhesin of 33 and 24 kDa; we have named the latter as Lsa25 because Lsa24 has been already described (Barbosa et al., 2006)

^3^http://blast.ncbi.nlm.nih.gov/Blast.cgi/

### Distribution and expression of LIC11834 and LIC12253 genes among *Leptospira* strains

**Figure 1:**
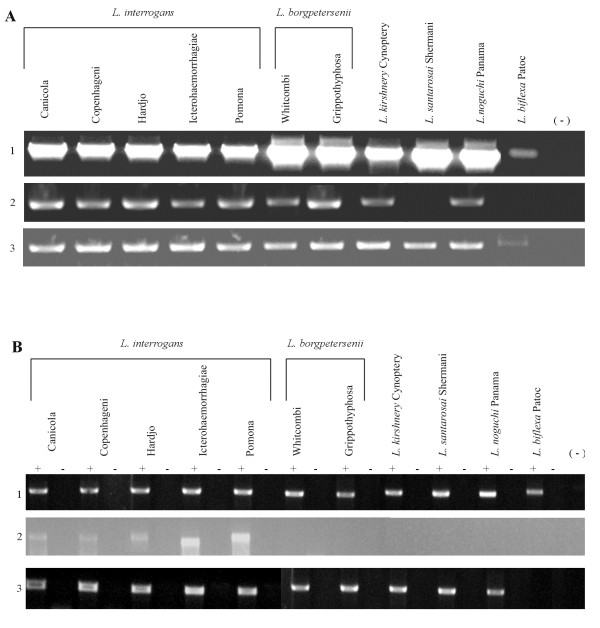
**Analysis of the LIC11834 and LIC12253 genes and their transcripts among different leptospiral strains.(A)** Analysis by PCR of the LIC11834 (2) and LIC12253 (3) genes in pathogenic serovars *(L. interrogans*, *L.borgpetersenii, L. kirshnery, L. noguchi andL. santarosai*) and in the non - pathogenic *L. biflexa* strain. 16 S rRNA gene expression was used as an internal control (1). The negative control contained no DNA, indicated by (−). **(B)** Analysis of the LIC11834 (2) and LIC12253 (3) transcripts by RT - PCR using 2 μg total RNA extracted from different serovars belonging to the pathogenic species and the saprophytic *L. biflexa* serovar Patoc strain Patoc. 16 S rRNA gene expression was used as an internal control (1). Reverse transcriptase present, +; reverse transcriptase omitted, -. The negative control contained no cDNA, indicated by (−).

The presence of LIC11834 and LIC12253 genes in pathogenic strains and in one saprophytic strain was examined by PCR with a pair of primers designed according to *L. interrogans* serovar Copenhageni genome sequences. The gene LIC11834 was amplified by PCR in all strains belonging to the pathogenic species excluding in *L. santarosai* serovar Shermani (Figure 1A). No DNA amplification was detected in the non - pathogenic *L. biflexa* serovar Patoc. In the case of LIC12253 gene, DNA band was amplified in all pathogenic strains and a less intense band was detected in the saprophytic strain (Figure 1A). The expression of LIC11834 and LIC12253 genes was evaluated by PCR amplification of reversely transcribed total RNA. LIC11834 gene product was detected only in *L. interrogans* specie serovars Canicola, Pomona, Copenhageni, Icterohaemorrhagiae and Hardjo. No expression was observed in non-pathogenic strain. LIC12253 gene expression could be identified in all pathogenic strain tested (Figure 1B). Integrity of total RNA used in RT - PCR experiments was assured by the presence of a 1,042 - bp 16 S ribosomal cDNA fragment in all samples (Figure 1B).

### Cloning and characterization of recombinant proteins

The amplified DNA sequences of LIC11834 and LIC12253 were cloned into an *E. coli* pAE vector [27] and the corresponding proteins were expressed as full-length with 6X His sequence tag at their N - terminal. Expression of recombinant proteins was elicited from cultures of *E. coli* BL21 SI after addition of NaCl (300 mM). Recombinant protein Lsa33 is expressed in its soluble form, while Lsa25 is expressed in its insoluble form, as inclusion bodies (data not shown). Protein Lsa25 was recovered from inclusion bodies after solubilization with 8 M urea. The purification was performed by metal chelating chromatography under normal (Lsa33) or denaturing condition, followed by refolding by gradually removal of urea (Lsa25). The proteins were recovered with 1.0 M imidazol. Evaluation of protein purification has shown that most of the contaminants were washed away and proteins were represented as single major bands. The recombinant protein bands were further confirmed by western blotting probed with monoclonal anti - His tag antibodies and with polyclonal antiserum raised against each protein (data shown). The calculated 33.1 kDa and 24.07 kDa molecular masses of the recombinant proteins comprise the vector fusion plus the encoded amino acids. Structural integrity of the purified proteins was assessed by circular dichroism (CD) spectroscopy. The minima at 208 and 222 nm, and the maximum at 192 nm in the CD spectrum showed the high α - helical secondary structure content of both recombinant proteins (data not shown).

### Recognition of the LIC11834 and LIC12253 coding sequences by immunofluorescence confocal microscopy

**Figure 2:**
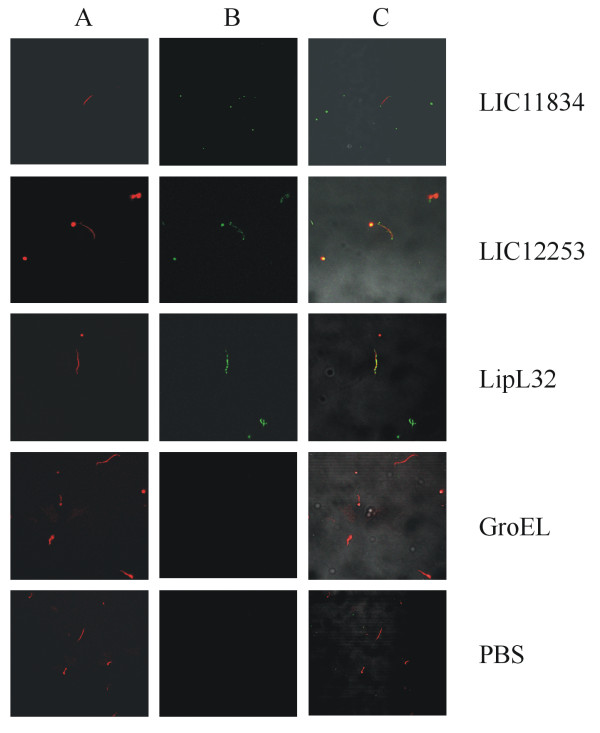
**Recognition of coding sequences LIC11834 and LIC12253 proteins in*L. interrogans*by their respective antibodies.** Liquid - phase Immunofluorescence Assay (L - IFA) was performed with live L. interrogans under confocal microscopy using antiserum for LIC11834, LIC12253, LipL32 (surface - exposed lipoprotein) and GroEL (protoplasmic cylinder marker). Leptospires were identified by propidium iodide staining of the DNA (**A**). FITC - conjugated secondary antibodies were used to detect the surface - bound antibodies (**B**). Co - localization is shown in the merged images (**C**).

The assessment of the selected CDSs on the bacterial cell membrane was performed using living organisms and the liquid - phase immunofluorescence method. Leptospires were visualized by propidium iodide staining (Figure 2, column A) followed by protein detection with polyclonal mouse antiserum raised against each protein in the presence of anti - mouse IgG antibodies conjugated to FITC. Green fluorescence could be observed in Figure 2 column B, for LIC11834, LIC12253 and LipL32, an outer membrane protein used as a positive control [28], but not with GroEL, a protoplasmic - cylinder marker, used as a negative control [29]. The localization of the protein - green light lying on the leptospires was achieved by merging both fields and the results obtained are shown in Figure 2, column C.

### Cellular localization of the LIC11834 and LIC12253 coding sequences by protease assay

**Figure 3:**
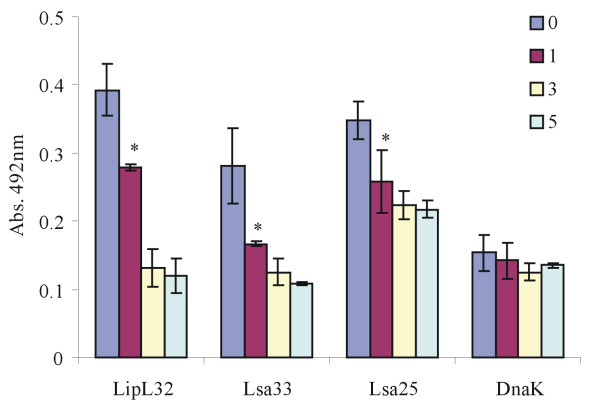
**Protease accessibility assay of LIC11834 and LIC12253 encoded proteins of*L. interrogans.*** Viable leptospires were incubated with 25 μg/ml of proteinase K at the indicated times. The suspensions were sedimented, washed, ressuspended in PBS and coated in a microplate. Antibodies against recombinant proteins Lsa33, Lsa25, LipL32 and DnaK were added. After incubation, anti-IgG peroxidase conjugated was added and the reaction was developed with OPD peroxidase substrate. Blanks were run in parallels but antibodies against the proteins were omitted. Readings were taken at 492 nm. Bars represent the mean of absorbance ± the standard deviation of three replicates for each protein and are representative of three independent experiments. For statistical analyses, the signal was compared between 0 hour and hours of treatment with PK by two-tailed *t* test (*P < 0.05).

We have performed proteinase K accessibility assay by using the previously described assay (37, 41) with some modifications. Live leptospires were treated with 25 μg/ml of proteinase K and aliquots of the bacterial suspensions were taken at time 0, 1, 3 and 5 h; the suspensions were sedimented and the ressuspended bacteria were used to coat microplates, followed by incubation with polyclonal antibodies against each protein, including the controls, LipL32 and DnaK, for outer [28] and cytoplasmic [30] protein. The reactions proceeded as described in Methods. The leptospiral coding sequences LIC11834 and LIC12253 were both susceptible to protease treatment after 1 h incubation, similar to the positive control LipL32 (Figure 3). Almost no reduction was observed with DnaK cytoplasmic protein (Figure 3).

### Recombinant protein Lsa25 is recognized by antibodies of confirmed cases of leptospirosis

**Figure 4:**
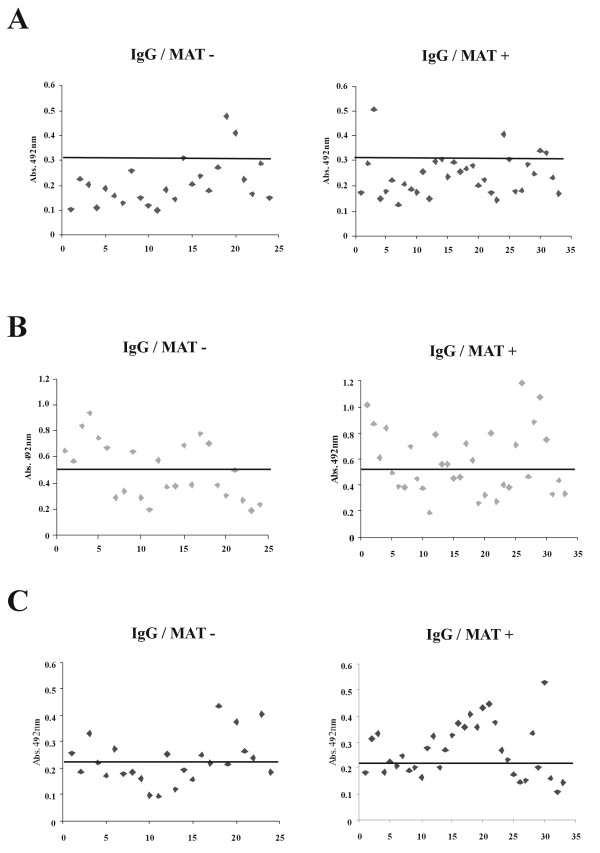
**Reactivity of the recombinant antigens Lsa33 and Lsa25 with serum samples of individuals diagnosed with leptospirosis. ** Responders were determined by ELISA with the recombinant proteins and serum samples from patients of both phases of the disease. The reactivity was evaluated as IgG antibodies. Serum was considered MAT positive or MAT negative if agglutination was detected when the sera were tested for their reactivity’s with isolates of the 22 serovars (see Methods). The cutoff values are depicted as horizontal bars and were defined as the mean plus 3 standard deviations obtained for sera from 12 healthy individuals. (**A**) shows the data for Lsa33, (**B**) for Lsa25, and (**C**) depicts the data when both proteins were employed (Lsa33 plus Lsa25).

To examine whether LIC11834 and LIC12253 leptospiral coding sequences are able to elicit an immune response from an infected host, we evaluated the reactivity of the recombinant proteins Lsa25 and Lsa33 with antibodies present in serum samples of early (MAT -) and convalescent (MAT +) phases of leptospirosis patients. ELISA was performed using 24 and 33 serum samples of negative MAT and of positive MAT, respectively. The recombinant protein Lsa33 was almost non-reactive with samples from both phases of the disease (Figure 4A), while Lsa25 showed 46 and 48% reactivity for negative and positive MAT, respectively (Figure 4B). When the two proteins were assayed together, a small increment was observed for positive MAT samples (58%) (Figure 4C). Our data suggest that Lsa25 might be an interesting protein for early diagnose of leptospirosis.

### Recombinant proteins adhesion to ECM components

**Figure 5:**
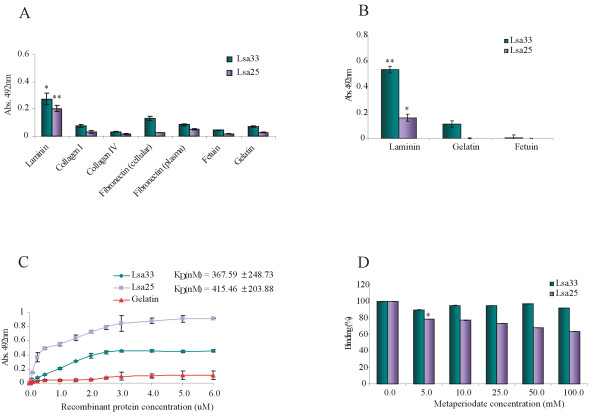
**Binding characteristics of Lsa33 and Lsa25 proteins to ECM components. (A)** Wells were coated with 1 μg of laminin, collagen type I, collagen type IV, cellular fibronectin, plasma fibronectin and the control proteins gelatin and fetuin. One μg of the recombinant proteins Lsa33 and Lsa25 was added per well and the binding was measured by ELISA. In **(A)** the protein binding was detected by polyclonal antibodies against each protein, while in **(B)** protein binding was evaluated with monoclonal anti - polyhistidine serum. Data represent the mean ± the standard deviation from three independent experiments. For statistical analyses, the attachment of recombinant proteins to the ECM components was compared to its binding to gelatin by the two - tailed *t* test (*P < 0.05). **(C)** Dose - dependent binding experiments of recombinant proteins with laminin was performed by polyclonal antibodies against each protein; each point was performed in triplicate and expressed as the mean absorbance value at 492 nm ± standard error for each point. Gelatin was included as a negative control. The dissociation constants (K_D_) are depicted and were calculated based on ELISA data for the recombinant proteins that reached equilibrium. **(D)** Immobilized laminin was treated with sodium metaperiodate (5 to 100 mM) for 15 min at 4°C in the dark. Mean absorbance values at 492 nm (± the standard deviations of three independent experiments) were compared to those obtained with untreated laminin (0 mM).

The ability of Lsa33 and Lsa25 proteins to mediate host colonization by adhering to extracellular matrix proteins was examined by ELISA. Laminin, collagen Type I, collagen Type IV, cellular fibronectin, plasma fibronectin, and the control proteins fetuin and gelatin were immobilized on microdilution wells and recombinant protein attachment was assessed by ELISA using antibodies against the proteins. As shown in Figure 5A, both recombinant proteins exhibited adhesiveness to laminin, while no statistically significant binding was observed with these proteins when wells were coated with collagen Type I and IV, cellular and plasma fibronectin, gelatin or with the highly glycosylated control protein fetuin. The interaction of recombinant proteins with laminin was also observed when anti - polyhistidine monoclonal antibodies were employed to probe the ligands (Figure 5B). The binding between Lsa33 and Lsa25 with laminin was also evaluated on a quantitative basis as depicted in Figure 5C. A dose - dependent and saturable binding was observed when increasing concentrations of the recombinant proteins (0–6 μM) were allowed to adhere to a fixed laminin concentration (1 μg) (Figure 5C). Binding saturation level was achieved by protein concentration of ~4 and 5 μM for Lsa33 and Lsa25, respectively. Based on ELISA data, the calculated dissociation equilibrium constants (*K*_D_) for the recombinant protein Lsa33 and Lsa25 with laminin is 367.5 and 415.4 nM, respectively. The role of laminin sugar moieties in the binding with Lsa33 and Lsa25 was assessed with laminin previously oxidized by increasing concentrations of sodium metaperiodate, ranging from 5 to 100 mM. The effect of metaperiodate concentration on the interaction is displayed in Figure 5D. Laminin oxidation had some effect on the interaction with Lsa25, being the reduction of 40% achieved at the highest metaperiodate concentration employed (100 mM). However, the attachment of Lsa33 to metaperiodate - treated laminin had no interference on the binding. These results indicate that the binding of the proteins to laminin occur in a different manner and that sugar residues contribute to some extent for the interaction of Lsa25 with this ECM glycoprotein. 

### Interaction of recombinant proteins to serum components

**Figure 6:**
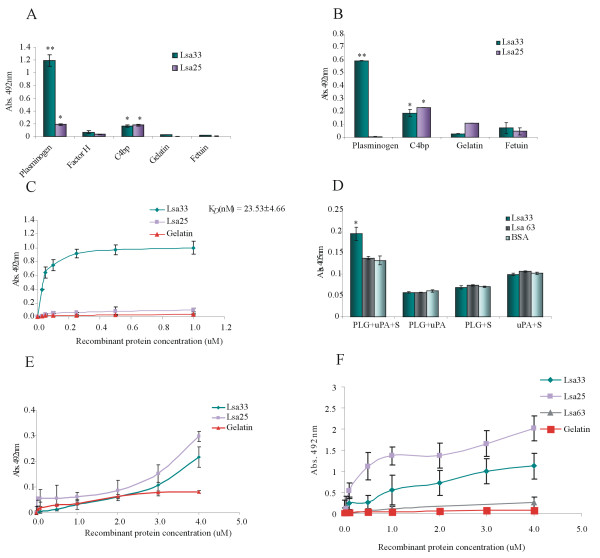
**Recombinant proteins binding to serum components. (A)** Human purified PLG, factor H and C4bp (10 μg/ml) were coated onto ELISA plates and allowed to interact with the recombinant proteins Lsa33 and Lsa25 (10 μg/ml). Gelatin and fetuin were used as negative controls for nonspecific binding. The binding was detected by antibodies raised against each recombinant protein (1:750). Bars represent the mean of absorbance at 492 nm ± the standard deviation of three replicates for each protein and are representative of three independent experiments. For statistical analyses, the binding of Lsa33 and Lsa25 was compared to its binding to gelatin by two - tailed *t* test (*P < 0.05 and **P < 0.005). **(B)** Similar as described in **(A)** but the binding of the recombinant proteins was detected by anti - polyhistidine monoclonal antibodies (1:200). Included is a His - tag recombinant protein Lsa63 that does not bind C4bp. **(C)** Recombinant proteins dose - dependent binding experiments with PLG. The binding was detected by polyclonal antibodies against each protein; each point was performed in triplicate and expressed as the mean absorbance value at 492 nm ± standard error for each point. Gelatin was included as a negative control. The dissociation constant (K_D_) is depicted and was calculated based on ELISA data for the recombinant protein that reached equilibrium. **(D)** Plasmin generation by PLG bound to recombinant proteins was assayed by modified ELISA as immobilized proteins received the following treatment: PLG + uPA + specific plasmin substrate (PLG + uPA + S), or controls lacking one of the three components (PLG + uPA; PLG + S; uPA + S). Lsa63 and BSA were employed as negative controls. Bars represent mean absorbance at 405 nm, as a measure of relative substrate degradation ± the standard deviation of four replicates for each experimental group and are representative of three independent experiments. Statistically significant binding in comparison to the negative control (BSA) are shown: *P < 0.05. **(E)** Recombinant proteins dose - dependent binding experiments with C4bp. The binding was detected by polyclonal antibodies raised against each protein (1:750); each point was performed in triplicate and expressed as the mean absorbance value at 492 nm ± standard error for each point. Gelatin was included as a negative control. **(F)** Similar to **(E)** but the protein binding was detected by anti-polyhistidine monoclonal antibodies (1:200); included is a His - tag recombinant protein Lsa63 that does not bind C4bp; each point was performed in triplicate and expressed as the mean absorbance value at 492 nm ± standard error for each point. Gelatin was included as a negative control.

Our group has recently reported that leptospires interact with PLG and that several proteins could act as PLG - receptors [17-19,21]. Protein binding to complement regulators factor H and C4bp have also been shown [31,32]. Therefore, we set out to evaluate whether the recombinant proteins Lsa33 and Lsa25 were capable of binding human PLG, factor H and C4bp *in vitro*. The components, human PLG, factor H and C4bp and the control proteins, gelatin and fetuin, were individually immobilized onto 96 - wells plates followed by incubation with the recombinant leptospiral proteins. The results obtained using polyclonal antibodies against each protein to probe the reactions showed that Lsa33 and Lsa25 interact with C4bp while only Lsa33 appears to bind to PLG (Figure 6A). No reaction was observed with factor H and the control proteins (Figure 6A). Similar results were achieved when binding was performed using monoclonal anti - his tag antibodies (Figure 6B). Both data show that while Lsa33 protein depicted a statistically significant absorption value for the interaction with PLG, the Lsa25 appears to have only a weak or no adherence to this component. These data were further confirmed when the reaction between the recombinant proteins and PLG were assessed on a quantitative basis as illustrated in Figure 6C. Dose - dependent and saturable binding was observed when increasing concentrations (0 to 1.0 μM) of the recombinant protein Lsa33 were allowed to adhere to a fixed PLG amount (1 μg), while very low absorption was detected with Lsa25 (Figure 6C). Based on the ELISA data, the calculated *K*_D_ for the recombinant proteinLsa33 with PLG is 23.53 ± 4.66 nM (Figure 6C). This *K*_D_ value is in the same order of magnitude with the ones obtained with several recombinant proteins in our laboratory [21].

PLG bound to leptospires and to several recombinant proteins, acting as PLG receptor, can acquire proteolytic activity in the presence of an activator, as we have previously shown [17-19,21]. Therefore, we investigated whether Lsa33 bound to PLG could also generate the enzymatically active plasmin. As a negative control, we have included the recombinant protein Lsa63, previously shown to be non-reactive with PLG [21]. Microplates were coated with the test protein, blocked, and then incubated with PLG. Unbound PLG was washed away and the urokinase - type PLG activator (uPA) was added together with a plasmin - specific chromogenic substrate. The reaction was carried out overnight and the plasmin activity was evaluated by measuring the cleavage of the substrate (absorbance at 405 nm). As shown in Figure 6D, the PLG captured by the Lsa33 protein could be converted into plasmin, as demonstrated indirectly by specific proteolytic activity. The negative controls Lsa63 and BSA did not show any proteolytic activity, similar to the controls lacking PLG, uPA or the chromogenic substrate.

The interaction of recombinant proteins with C4bp was studied in function of protein concentration. We have employed anti –Lsa33 and anti-Lsa25 polyclonal (Figure 6E) and anti-His tag monoclonal antibodies (Figure 6F) to probe the binding. Dose - response curves were obtained with both antibodies but the best response was achieved with anti-His tag monoclonal (Figure 6F), probably because of their homogeneous nature. However, C4bp was not saturated with the protein concentration range employed and therefore the *K*_D_ could not be calculated. Lsa63, a His - tag recombinant protein that does not bind C4bp was also included, as a negative control, showed very low interaction and did not respond to increase protein concentration.

### Inhibition of *L. interrogans* attachment to laminin or to PLG by Lsa33 and Lsa25

**Figure 7:**
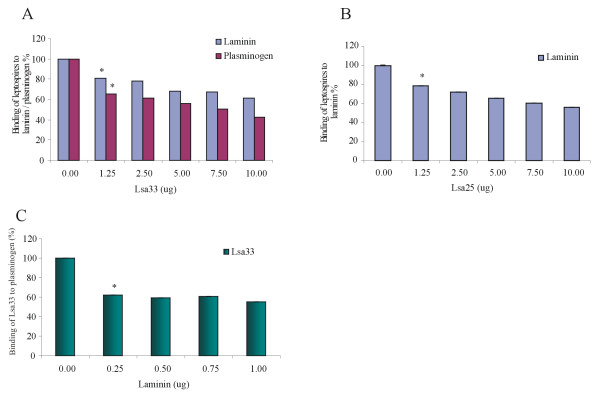
**Inhibition of*L. interrogans*attachment to immobilized laminin and PLG by recombinant proteins; The effect of laminin concentration on the binding of PLG to Lsa33**. (**A**) Laminin or PLG (1 μg/well) was adsorbed onto microtiter plates followed by incubation with increasing concentrations of Lsa33 (0 to 10 μg) and in (**B**) laminin was adsorbed onto microtiter plates followed by incubation with increasing concentrations of Lsa25 (0 to 10 μg). In (**A**) and (**B**) the incubations were allowed to proceed for 90 min at 37°C. Live leptospires (100 μl/well of 4 X 10^7^*L. interrogans* serovar Copenhageni strain M20 leptospires) were added and incubated for another 90 min at 37°C. The unbound leptospires were washed away, and the quantification of bound leptospires was performed indirectly by anti - LipL32 antibodies produced in mice (1: 4,000 dilution) followed by horseradish peroxidase - conjugated antimouse IgG antibodies. Each point represents the mean absorbance value at 492 nm ± standard deviation of three replicates. Data are representative of two independent experiments (*P < 0.05). (**C**) The effect of laminin on the binding of PLG (10 μg/ml) to immobilized rLIC11834 (10 μg/ml) was assessed with the addition of increasing concentrations of laminin (0 to 1.0 μg). The detection of rLIC11834-bound PLG was performed by use of specific antibodies anti - PLG. Bars represent the mean absorbance values ± standard deviation of four replicates for each condition and are representative of two independent experiments. Results of statistically significant interference on the binding in comparison with the control (no addition of laminin) are shown: *P < 0.05.

It has been reported that the several recombinant proteins with adhesin activity revealed an inhibitory effect on the binding of leptospires to ECM macromolecules [6]. We therefore performed experiments to assess whether the recombinant proteins had an effect on the binding of *Leptospira* to laminin or PLG by employing ELISA to detect the interaction in function of protein concentration (0–10 μg). The results demonstrate that the addition of increasing concentrations of Lsa33 reduced the leptospiral binding to laminin and to PLG molecules in a dose - dependent manner (Figure 7A). Binding decrease in the number of leptospires interacting to laminin and PLG was statistically significant with 1.25 μg of Lsa33 (*, *P* < 0.05). This interference was also evaluated with the binding of leptospires to laminin in the presence of increasing concentrations of Lsa25 (0–10 μg), resulting in a similar effect as obtained with Lsa33 (*, *P* < 0.05) (Figure 7B). We have also assessed the competition for the binding of Lsa33 to PLG by increasing laminin concentration (0 to 1.0 μg). The results revealed that both ligands compete for the binding with Lsa33 as a decrease of 40% in the binding was already detected with 0.25 μg of laminin (*, *P* < 0.05) (Figure 7C). These experiments were performed in triplicate and Figure 7 shows one representative data of two independent experiments.

## Discussion

Complement is a key component of the innate immune system responsible for protection against pathogenic microorganisms [33]. Factor H is a host fluid - phase regulator of the alternative complement pathway. Pathogenic leptospiral complement - resistant strains were found to bind factor H from human serum and this interaction seems to be associated to their serum resistance [31,34]. C4b - binding protein is an inhibitor of complement classical pathway system. This protein controls the complement classical pathway by interfering with the formation and regeneration of C3 convertase and acting as a cofactor to the serine proteinase factor I in the proteolytic inactivation of C4b [33,35]. It has been shown that pathogenic leptospiral strains can obtain C4bp from the host and that this acquisition preserves its cofactor activity [36]. The surface bound C4bp elicits factor I mediated cleavage of C4b, an interaction that appears to contribute to complement resistance by *Leptospira*, through the classical route [37]. Leptospiral binding proteins to C4bp, factor H and factor H - like have also been identified in *Leptospira*[9,31,32]. Interaction of C4bp and of factor H with other pathogens has been described, including the spirochetes *Borrelia* spp. [33,37-41].

The capacity of the leptospires to adhere to extracellular matrix components has been reported and to date, several leptospiral adhesins have been identified. These include 36 - kDa fibronectin - binding protein [42], LfhA/Lsa24 [6,31], LigA and LigB proteins [7,8], Len-family proteins [9], Lsa21 [10], LipL32 [12,43], Lsa27 [13], Lp95 [11], TlyC [14], LipL53 [44], Lsa63 [15], OmpL37 [45], Lsa66 [17] and Lsa20 [18]. We have reported that *Leptospira* species were also capable to bind PLG and generating plasmin, in the presence of host activator, on the outer surface *in vitro*[19]. In addition, we have described that plasmin - coated virulent *L.interrogans* bacteria were capable to degrade purified extracellular matrix components fibronectin [19] and laminin (Vieira et al., unpublished data), a step that may contribute for dissemination of the bacteria through the host tissues. More recently, we have shown that plasmin generation on the bacterial surface decreases the deposition of C3b and IgG and hence, opsonization and phagocytosis, a process that could facilitate leptospires to evade the immune system [22]. Several PLG-receptor proteins in *Leptospira* have been identified [17,18,20,21].

By data mining the genome sequences of *L. interrogans*, searching for surface exposed proteins that could mediate host - pathogen interactions, we have identified two proteins annotated as *Leptospira* conserved hypothetical, one of them, predicted to be a novel lipoprotein, LIC11834, and the other, LIC12253, has recently been shown to be non-protective in leptospiral challenge assay [46]. Both selected coding sequences were cloned and the recombinant proteins expressed in *E. coli*. We report that these proteins, Lsa33 and Lsa25, are laminin - binding adhesins and in the case of Lsa33, capable to bind PLG generating enzymatically active plasmin. Although weak, both proteins showed the ability to bind human purified C4bp, suggesting that these proteins have the potential to participate in leptospiral immune evasion by interfering with the complement classical pathway.

Due to the high degree of antigenic variation among leptospires, we examined the gene/protein conservation among important species of *Leptospira*. The LIC11834 and LIC12253 genes are conserved in five serovars of *L. interrogans* and in other species tested but in the case of *L. santarosai* serovar Shermani the gene LIC11834 is absent. However, LIC11834 transcripts were detected only in serovars of *L. interrogans*, while LIC12253 appears to be expressed in all strains evaluated. None of the proteins seems to be expressed in the saprophytic strain, *L. biflexa* serovar Patoc.

The recombinant proteins Lsa33 and Lsa25 were expressed with molecular mass of 33 - and 24 - kDa, respectively, having a 6XHis tag at N - terminal. The purified proteins exhibited single major bands in SDS - PAGE and were recognized by anti - His tag monoclonal antibodies and by homolog sera from mice immunized with each recombinant protein. Secondary structure of the recombinant proteins after the purification process was evaluated by CD spectroscopy and showed a predominance of alpha helices in both cases, similar to the data predicted by bioinformatics, indicating the suitability of recombinant proteins for further studies. The LIC12253 coding sequence is probably higher immunogenic than LIC11834 because it was recognized by approximately 45% of serum samples of both phases, initial and convalescent, of confirmed leptospirosis’s cases. Interestingly, the LIC11834 protein although presented almost no reactivity among these serum samples, showed a slightly augment effect on serum reactivity when was assayed together with LIC12253. Immunofluorescence using live leptospires showed LIC11834 and LIC12253 coding sequences at the surface of bacteria, as a result of antiserum recognition raised against each protein. *In silico* analysis, proteinase K accessibility and immunofluorescence data together suggest that these proteins are likely to be surface exposed. In addition, the recombinant proteins partially inhibited leptospiral adherence to immobilized laminin and PLG.

Merien and colleagues [42] identified a 36-kDa fibronectin-binding protein expressed by a virulent variant of *Leptospira*. Our group described the first leptospiral laminin - binding protein, named Lsa24 [6]. These studies were followed by the identification of several extracellular matrix binding proteins [7,9-18]. The recombinant proteins Lsa33 and Lsa25 exhibited extracellular matrix - binding properties, and are laminin - binding proteins. The binding affinity dissociation constants estimated for both proteins to laminin showed similar K_*D*_ value of that reported for OmpL 37 (410 ± 81 nM) and the same ECM molecule [16]. Thus, it is possible that these proteins have a role in the adhesion of leptospires to hosts.

The PLG activation system with generation of plasmin was described for virus, parasites and bacteria, including the spirochetes *Borrelia* spp. and with *Treponema denticola*[47-50]. Plasmin is a serine protease with the capacity to degrade a broad spectrum of substrates, including fibrin clots, connective tissue and components of extracellular matrices [51-53]. We have reported that *Leptospira* spp. bind PLG at their surface generating plasmin, when host activator is available, making the bacteria capable to degrade fibronectin [19] and laminin (Vieira, M.L., unpublished results). Verma et al. [20] have demonstrated that the protein LenA of *L. interrogans*[9] is a surface receptor for human PLG. Moreover, we have reported several novel PLG - receptor proteins of *Leptospira*[17,18,21]. We now describe Lsa33 as a novel PLG - binding protein. Similar to the previously reported proteins, bound PLG could be converted to plasmin by the addition of urokinase - type PLG activator (uPA), showing specific proteolytic activity. It is thus possible that the Lsa33 besides playing a role in the attachment to host and acting as PLG - receptor, may also help leptospires to surmount tissue barriers.

The inhibitory effect exerted on the binding of leptospires to laminin and PLG by the recombinant proteins was statistically significant with both, in the case of Lsa33, and with laminin for the Lsa25. The intensity of the interference upon the binding is expected given the presence of several ECM - or PLG-binding proteins in *Leptospira*. These data are comparable to the ones already reported in the literature [6,7,16-18,21]. Partial inhibitory effect was observed by laminin on the binding of Lsa33 to PLG, suggesting a competition for the same binding site.

## Conclusions

We report in these studies a characterization of two leptospiral proteins, genome annotated as proteins of unknown function. The recombinant proteins Lsa33 and Lsa25 are laminin binding proteins that might be involved in the attachment to host. Moreover, both proteins showed the ability to bind C4bp, a feature suggesting their possible involvement in the immune evasion of leptospires. The recombinant Lsa33 is also PLG - binding protein that could help the bacteria during the infection process. Thus, it appears that Lsa33 and to a lesser degree, Lsa25, are multifaceted proteins that might have multiple functions in the leptospiral pathogenesis. To date, Lsa33 is the first described laminin -, PLG - and C4bp - leptospiral binding protein.

## Methods

### *Leptospira* strains and sera

The pathogenic *Leptospira* strains used were: *L. interrogans* serovar Canicola strain Hound Utrech IV, *L. interrogans* serovar Copenhageni strain M 20, *L. interrogans* serovar Hardjo strain Hardjoprajitno, *L. interrogans* serovar Icterohaemorrhagiae strain RGA, *L. interrogans* serovar Pomona strain Pomona, *L. borgpetersenii* serovar Whitcombi strain Whitcomb and serovar Grippothyphosa strain Moskva V, *L. kirshneri* serovar Cynoptery strain 3522 C, *L. santarosai* serovar Shermani strain 1342 K, *L. noguchi* serovar Panama strain CZ 214 and *L. biflexa* serovar Patoc strain Patoc, were cultured at 28°C under aerobic conditions in liquid EMJH medium (Difco®) with 10% rabbit serum, enriched with L - asparagine (wt/vol: 0.015%), sodium pyruvate (wt/vol: 0.001%), calcium chloride (wt/vol: 0,001%), magnesium chloride (wt/vol: 0.001%), peptone (wt/vol:0.03%) and meat extract (wt/vol: 0.02%) (Turner LH. *Leptospirosis. 3. Maintenance, isolation and demonstration of leptospires*. Trans R Soc Trop Med Hyg 1970; 64: 623–646). *Leptospira* cultures are maintained in Faculdade de Medicina Veterinária e Zootecnia, USP, São Paulo, SP, Brazil. Confirmed - leptospirosis serum samples were from Instituto Adolfo Lutz collection, São Paulo, Brazil.

### Microscopic agglutination test (MAT)

The microscopic agglutination test was performed according to [1]. In brief, an array of 22 serovars of *Leptospira* spp. as antigens were employed: Australis, Autumnalis, Bataviae, Canicola, Castellonis, Celledoni, Copenhageni, Cynopteri, Djasiman, Grippotyphosa, Hardjo, Hebdomadis, Icterohaemorrhagiae, Javanica, Panama, Patoc, Pomona, Pyrogenes, Sejroe, Shermani, Tarassovi and Wolffi. All the strains were maintained in EMJH liquid medium (Difco, USA) at 29°C. A laboratory - confirmed case of leptospirosis was defined by the demonstration of a four - fold microagglutination titer rise between paired serum samples. The probable predominant serovar was considered to be the one with the highest dilution that could cause 50% of agglutination. MAT was considered negative when the titer was below 100.

### Characterization of the protein *in silico*

Predicted coding sequence (CDSs) LIC11834 and LIC12253 were identified on *L. interrogans* serovar Copenhageni and selection was based on cellular localization; cellular localization prediction was performed by PSORT, http://psort.nibb.ac.jp[54] and PredictProtein web server, https://www.predictprotein.org/[25]. The SMART [23]http://smart.embl-heidelbergde/ and PFAM [55]http://www.sanger.ac.uk/Software/Pfam/ web servers were used to search for predicted functional and structural domains. The presence of lipobox putative sequence was evaluated by use of the LipoP program [56]http://www.cbs.dtu.dk/services/LipoP/. The predicted sequence of the lipobox was also assessed by use of the SpLip program, as described by Setubal et al. [57]. Secondary structure, solvent accessibility and cellular localization predictions were also performed by using PredictProtein web server, https://www.predictprotein.org/[25].

### DNA isolation and PCR analysis

*Leptospira* cultures were harvested by centrifugation at 11,500 g for 30 min and gently washed in sterile PBS twice. Genomic DNA was isolated from the pellets by guanidine - detergent lysing method using DNAzol® Reagent (Invitrogen), according to the manufacturer’s instructions. Primers were designed according to *L. interrogans* serovar Copenhageni genome sequences (GenBank accession AE016823) and are listed in Table 1. PCR was performed in a reaction volume of 25 μl containing 100 ng of genomic DNA, 1 × PCR buffer (20 mM Tris - HCl, pH 8.4, 50 mM KCl), 2 mM MgCl_2_, 20 pmol of each specific primer, 200 μM of each dNTP, and 2.5 U Taq DNA Polymerase (Invitrogen). Cycling conditions were: 94 ° C - 4 min, followed by 40 cycles at 94°C - 50 sec, 57°C (LIC11834) or 56°C (LIC12253) - 50 sec, 72°C - 90 sec, and a final extension cycle of 7 min at 72°C. PCR amplified products were loaded on a 1% agarose gel for electrophoresis and visualization with ethidium bromide.

### RNA extraction and RT-PCR analysis

For reverse transcription RT - PCR, total RNA was isolated by the acid guanidinium thiocyanate phenol - chloroform method using TRIzol® Reagent (Invitrogen) according to the manufacturer’s recommendations. 1 μg of RNA from each sample was treated with 1 U of DNAse I Amplification Grade (Invitrogen) for 15 min at room temperature. DNAse I was inactivated by the addition of 1 μl of 25 mM EDTA solution followed by an incubation at 65 ° C for 10 min. DNAse - treated RNAs were reversely transcribed using the SuperScript™ III First - Strand Synthesis System for RT-PCR (Invitrogen). One tenth of RT products were amplified in a 25 μl reaction mix using oligonucleotides LIC11834 - F/LIC11834 - R or LIC12253 - F/LIC12253 - R, as described above. Samples quantity and integrity were verified by amplification of a 1,042 bp 16 S ribosomal cDNA fragment using oligomers:

16S - F 5′CAAGTCAAGCGGAGTAGCAATACTCAGC 3′ and 16S - R 5′GATGGCAACATAAGGTGAGGGTTGC 3′.

### DNA recombinant techniques, protein expression and purification

Predicted CDSs LIC11834 and LIC12253, without signal peptides, were amplified by the PCR from *L. interrogans* serovar Copenhageni strain Fiocruz L1 - 130 genomic DNA using the primer pairs depicted in Table 1. The PCR products obtained for each corresponding gene were cloned into pGEM-T easy vector (Promega) and subcloned into the pAE expression vector [27] at the restriction sites shown in Table 1. The pAE vector allows the expression of recombinant proteins with a minimal 6X His - tag at the N - terminus. All cloned sequences were confirmed by DNA sequencing with an ABI 3100 automatic sequencer (PE Applied Biosystems, Foster city, CA). Protein expression of rLIC11834 and rLIC12253 was achieved in *E. coli* BL21 (SI) strain by the action of T7 DNA polymerase under control of the osmotically induced promoter proU [58]*. E. coli* BL21 (SI) containing recombinant plasmids were grown at 30°C in Luria - Bertani broth without NaCl and with 100 μg/ml ampicillin with continuous shaking until an optical density at 600 nm of 0.6 to 0.8 was reached. Recombinant protein synthesis was induced by the addition of 300 mM NaCl. After three hours, the cells were harvested by centrifugation and the bacterial pellets resuspended in lysis buffer (200 μg/ml of lysozyme, 1% Triton X - 100, 2 mM phenylmethylsulfonyl fluoride [PMSF]). The bacterial cell pellets were lysed on ice with the aid of a sonicator (Ultrasonic Processor; GE Healthcare). The insoluble fractions were washed with 20 ml of buffer (20 mM Tris - HCl, pH 8.0, 500 mM NaCl, 1 M urea and 1% Triton X-100) and resuspended in a buffer containing 20 mM Tris - HCl, pH 8.0, 500 mM NaCl, 5 mM Imidazole, 1 mM β - mercaptoethanol and 8 M urea. The proteins were then purified through metal chelating chromatography in a Sepharose fast flow column (GE Healthcare) and fractions were analyzed in 12% SDS-PAGE. The rLIC12253 protein was refolded by 500 times dilution with 20 mM Tris - HCL, pH 8.0, and 500 mM NaCl before chromatographic purification. The purified recombinant proteins were extensively dialyzed against phosphate - buffered saline (PBS), pH 7.4, 0.1% (wt/vol) glycine solution (1:100), pooled and stored at −20°C.

### Circular dichroism spectroscopy

Purified recombinant proteins were dialyzed against sodium phosphate buffer (pH 7.4). Circular dichroism (CD) spectroscopy measurements were performed at 20°C using a Jasco J-810 spectropolarimeter (Japan Spectroscopic, Tokyo) equipped with a Peltier unit for temperature control. Far-UV CD spectra were measured using a 1 mm - path - length cell at 0.5 nm intervals. The spectra were presented as an average of five scans recorded from 185 to 260 nm. The molar ellipticity (Φ) is expressed in deg.cm.dmol^1^.

### Antiserum

Five female BALB/c mice (4–6 weeks old) were immunized subcutaneously with 10 μg of the recombinant proteins. The recombinant proteins were adsorbed in 10% (vol/vol) of Alhydrogel (2% Al(OH)_3_, Brenntag *Biosector*, Denmark), used as adjuvant. Two subsequent booster injections were given at two - week intervals with the same preparation of 10 μg of the proteins*.* Negative - control mice were injected with PBS. One week after each immunization, the mice were bled from the retro - orbital plexus and the pooled sera were analyzed by enzyme -linked immunosorbent assay (ELISA) for determination of antibody titers. All animal studies were approved by the Ethics Committee of the Instituto Butantan, São Paulo, SP, Brazil. The Committee in Animal Research in Instituto Butantan adopts the guidelines of the Brazilian College of Animal Experimentation.

### Immunoblotting assay

The purified recombinant proteins were loaded into 12% SDS - PAGE and transferred to nitrocellulose membranes (Hybond ECL; GE Healthcare) in semi - dry equipment. Membranes were blocked with 5% non-fat dried milk and 2.5% BSA in PBS containing 0.05% Tween 20 (PBS - T) and then incubated with anti - rLIC11834 (1:500), anti - rLIC12253 (1:500) mouse serum or anti - his antibody (1:1,000) (GE Healthcare) for 2 h at room temperature. After washing, the membranes were incubated with horseradish peroxidase (HRP) - conjugated anti - mouse IgG (1:5,000; Sigma) in PBS - T for 1 h. The protein’s reactivity was revealed by ECL reagent kit chemiluminescence substrate (GE Healthcare) with subsequent exposition to X - Ray film.

### ELISA for detection of human antibodies

Human IgG antibodies against Lsa33 or Lsa25 were detected by ELISA as previously described [59]. In brief, serum samples of negative (24) and positive (33) MAT from confirmed - leptospirosis patients were diluted 1:400 and evaluated for total IgG using goat HRP - conjugated anti-human IgG antibodies (1:5,000, Sigma). Cutoff values were set at three standard deviations above the mean OD_492nm_ of sera from 11 health individuals, unexposed to leptospirosis, from the city of São Paulo, Brazil and one pool of normal serum samples from USA (Sigma).

### Proteinase K accessibility assay

Suspensions of 5 ml live leptospires/per treatment (~10^8^cells/ml) were harvested at 10,000 rpm for 15 min at room temperature, washed once with PBS (with 50 mM NaCl), resuspended in 5 ml of PBS (with 50 mM NaCl) plus with 25 μg/ml of proteinase K (PK) (Sigma Aldrich). Four similar test tubes were then incubated for 0 to 5 h at 37°C and aliquots were taken at 0, 1, 3 and 5 h before the addition of 100 mM of phenylmethylsulfonyl fluoride (PMSF) to stop PK activity. The suspensions were subsequently pelleted by centrifugation at 10,000 rpm for 5 min, washed twice with PBS (with 50 mM NaCl) and resuspended in 1 ml PBS (with 50 mM NaCl) for ELISA analysis using antibodies against Lsa33, Lsa25, Lip32 and DnaK, as described below. LipL32 and DnaK are membrane and cytoplasmic leptospiral proteins that were employed in our experiment as positive and negative control, respectively.

### ELISA for detection cellular localization of the proteins

Leptospires were coated onto microplates and allowed to stand at room temperature for 16 h. The plates were washed three times with PBS (with 50 mM NaCl) and blocked with 5% non-fat dry milk and 1% BSA for 2 h at 37°C. After incubated for 2 h at 37°C with polyclonal mouse anti - serum against Lsa33, Lsa25, LipL32 or DnaK (dilution of an OD equal to 1). The leptospires were washed three times with PBS (with 50 mM NaCl) and incubated with 50 μL of a 1:5,000 dilution of HRP - conjugated goat anti - mouse IgG (Sigma) in PBS (with 50 mM NaCl) for 1 h at 37°C. The wells were washed three times with PBS (with 50 mM NaCl), and o - phenylenediamine (OPD) (1 mg/mL) in citrate phosphate buffer (pH 5.0) plus 1 μL/mL H_2_O_2_ was added (100 μL per well). The reaction proceeded for 5 min and was interrupted by the addition of 50 μL of 4 N H_2_SO_4_. The absorbance at 492 nm was determined in a microplate reader (TP - reader, Thermo) against the O.D. of blanks, containing all the reaction mixture but antibodies against the proteins. For statistical analyses, the binding of polyclonal mouse anti - serum against Lsa33, Lsa25, LipL32 or DnaK at 0 h incubation was compared with other incubations by Student’s two - tailed *t* test.

### Binding of recombinant proteins to ECM and to serum components

Protein attachment to individual macromolecules of the extracellular matrix was analyzed according to a previously published protocol [6] with some modifications. Briefly, 96 - well plates (Costar High Binding, Corning) were coated with 1 μg of laminin, collagen type I, collagen type IV, cellular fibronectin, plasma fibronectin, human PLG, factor H, C4bp, or gelatin (negative control) and fetuin (highly glycosylated attachment - negative control protein) in 100 μL of PBS for 3 h at 37°C. The wells were washed three times with PBS - T and then blocked with 200 μL of 10% (wt/vol) non-fat dry milk (overnight at 4°C). One microgram of each recombinant protein was added per well in 100 μL of PBS, and protein was allowed to attach to the different substrates for 2 h at 37°C. After washing six times with PBS - T, bound Lsa33 or Lsa25 was detected by adding mouse anti - recombinant proteins (1:750) in 100 μL of PBS or anti - His tag monoclonal (1:200) in 100 μL of PBS. Incubation proceeded for 1 h at 37°C. After three washings with PBS - T, 100 μL of a 1:5,000 dilution of HRP - conjugated goat anti - mouse IgG (Sigma) in PBS was added per well for 1 h at 37°C. The wells were washed three times, and o - phenylenediamine (OPD) (1 mg/mL) in citrate phosphate buffer (pH 5.0) plus 1 μL/mL H_2_O_2_ was added (100 μL per well). The reaction proceeded for 10 min and was interrupted by the addition of 50 μL of 4 N H_2_SO_4_. The absorbance at 492 nm was determined in a microplate reader (TP - reader, Thermo). For statistical analyses, the binding of recombinant proteins to ECM macromolecules and to serum components were compared to its binding to gelatin and by Student’s two - tailed *t* test.

### Metaperiodate treatment of laminin

Microtitre wells were coated with 1 μg of laminin in 50 mM sodium acetate buffer, pH 5.0, and incubated for 16 h at 4°C. Wells were washed three times with the same buffer, and laminin was treated with different sodium metaperiodate concentrations (0–100 mM) in the same buffer for 15 min at 4°C in the dark. After three washes with 50 mM sodium acetate buffer, wells were blocked with 200 μl of 1% BSA for 1 h at 37°C. Binding of recombinant proteins (1 μg in PBS per well) to periodate - treated laminin was evaluated as described above.

### Dose–response curves

First, 96 - well plates were coated overnight in PBS at 4°C with 100 μl of 10 μg/ml PLG, laminin or C4bp. Plates were then blocked and increasing concentrations of the purified recombinant proteins (0–6 μM) were added (100 μl/well in PBS). The assessment of bound proteins was performed by incubation for 1 h at 37°C with the antiserum raised against each protein at the dilution of 1:750, followed by HRP - conjugated goat anti-mouse IgG (Sigma) (1:5,000 in PBS). The reactions were detected with OPD as describe above. The ELISA data were used to calculate the dissociation constant (K_D_) according to the method described by Pathirana et al. [60] and Lin et al. [61], based on the equation: $A=A_{\text{max}}\left[\text{protein}\right]/\left(K_{D}+\left[\text{protein}\right]\right)$, where A is the absorbance at a given protein concentration, A_max_ is the maximum absorbance for the ELISA plate reader (equilibrium), [protein] is the protein concentration and K_D_ is the dissociation equilibrium constant for a given absorbance at a given protein concentration (ELISA data point).

### Plasmin enzymatic activity assay

96 - well ELISA plates were coated overnight with 10 μg/ml recombinant proteins in PBS at 4°C. Lsa63, which does not bind PLG [21] and BSA were employed as negative control. Plates were washed once with PBS - T and blocked for 2 h at 37°C with PBS with 10% (wt/vol) non - fat dry milk. The blocking solution was discarded and 100 μl/well of 10 μg/ml human PLG was added, followed by incubation for 2 h at 37°C. Wells were washed three times with PBS - T, and then 4 ng/well of human uPA (Sigma - Aldrich) were added. Subsequently, 100 μl/well of plasmin - specific substrate _D_ - valyl - leucyl – lysine - *p* - nitroanilide dihydrochloride (Sigma - Aldrich) were added at a final concentration of 0.4 mM in PBS. Plates were incubated overnight at 37°C and substrate degradation was measured by readings at 405 nm.

### Inhibition of live leptospires binding to laminin or to PLG by recombinant proteins

ELISA plates were coated with laminin or PLG (1 μg/well). The plates were washed and blocked with 10% non - fat dry milk in PBS - T for 2 h at 37°C. The blocking solution was discarded, and the wells were incubated for 90 min at 37°C with increasing concentrations of proteins (0 to 10 μg). After three washings, 50 μL/well of 4 × 10^7^ live low - passage virulent *L. interrogans* serovar Copenhageni strain M20 were added for 90 min at 37°C. The unbound leptospires were washed and the quantification of bound leptospires was performed indirectly by anti - LipL32 antibodies produced in mice (1:4,000), given the fact that LipL32 is a major outer membrane leptospiral protein [28]; the procedure was followed by horseradish peroxidase - conjugated anti - mouse IgG antibodies, essentially as described in Barbosa et al. [6]. The detection was performed by OPD as previously described.

### Liquid-phase immunofluorescence assay (L - IFA)

The localization of LIC11834 and LIC12253 encoded proteins by L - IFA was performed as described by Oliveira et al. [15]. In brief, suspensions of 2.5 ml live leptospires (~10^9^cells/ml) were harvested at 10,000 rpm for 15 min, washed twice with PBS (with 50 mM NaCl), resuspended in 200 μl of PBS with 6 μg/ml propidium iodide to stain the nuclei, and incubated for 45 min at 37°C. After incubation, the leptospires were washed gently with PBS and incubated for 30 min at 4°C with polyclonal mouse anti - serum against Lsa33, Lsa25, LipL32 or GroEL at a 1:50 dilution. The leptospires were washed and incubated with goat anti - mouse IgG antibodies conjugated to fluorescein isothiocyante (FITC, Sigma) at a dilution 1:50 for 30 min at 4°C. After incubation with secondary antibody, the leptospires were washed and resuspended in PBS - antifading solution (ProLong Gold, Molecular Probes). The immunofluorescence - labeled leptospires were examined by employ of a confocal LSM 510 META immunofluorescence microscope (Zeiss, Germany).

### Nucleotide sequence accession numbers

GenBank accession numbers for protein sequences LIC11834 and LIC12235 are AAS70420 and AAS70825, respectively. The protein can also be accessed by the genome nomenclature for the gene locus, LIC number (*Leptospira interrogans* serovar Copenhageni).

### ECM and biological components

The control proteins fetuin and gelatin, were purchased from Sigma Chemical Co. (St. Louis, Mo.) and Difco®, respectively. Laminin - 1 and collagen Type IV were derived from the basement membrane of Engelbreth - Holm-Swarm mouse sarcoma, cellular fibronectin was derived from human foreskin fibroblasts, plasma fibronectin was isolated from human plasma and collagen Type I was isolated from rat tail. PLG, purified from human plasma, was purchased from Merck. Human Factor H was from Calbiochem. C4bp was from Complement Technology, INC.

## Author’s contributions

RFD performed the molecular cloning studies, protein expression, ECM assays and animal immunizations. MLV carried out the PLG assays and help with the manuscript. ECR evaluated MAT of the collection serum samples. APG and ZMM were responsible for bacteria growth, identification and virulence strain maintenance. SAV participated in the design of the study and help drafted the manuscript. ALTON conceived of the study, and participated in its design and coordination and helped to draft the manuscript. All authors read and approved the final manuscript.
